# The double-edged sword of ovarian cancer information for women at increased risk who have previously taken part in screening

**DOI:** 10.3332/ecancer.2016.650

**Published:** 2016-06-30

**Authors:** Stephanie Smits, Jacky Boivin, Usha Menon, Kate Brain

**Affiliations:** 1 Divison of Population Medicine, School of Medicine, Cardiff University, Neuadd, Meirionnydd, Heath Park, Cardiff CF14 4YS, United Kingdom; 2 School of Psychology, Cardiff University, Cardiff CF10 3AT, United Kingdom; 3 Institute for Women’s Health, University College London, London W1T 7DN, United Kingdom

**Keywords:** ovarian cancer, symptoms, qualitative, family history, perceived risk

## Abstract

**Background:**

Women at increased risk who decide not to have, or to delay, risk-reducing salpingo-oophorectomy have to rely on early diagnosis through symptom awareness and presenting to primary care as soon as possible in the absence of screening. However, little is known about the acceptability to women of this strategy. We aimed to gain an in-depth understanding of women’s perceptions and previous experiences of ovarian cancer symptom management, and the influences on ovarian cancer awareness and anticipated symptom presentation.

**Method:**

Qualitative interviews were conducted with eight women at increased risk of ovarian cancer who had previously taken part in ovarian cancer screening and analysed using interpretative phenomenological analysis (IPA).

**Results:**

Familial experience of ovarian cancer and perceived personal risk shaped women’s perceptions and behavioural responses to disease threat. Ovarian cancer information was perceived to be a double-edged sword, regarded as either useful for increasing knowledge and confidence in discussing symptom concerns with health professionals or to be avoided due to fears about cancer.

**Conclusion:**

Women may be cautious about searching for information independently and in the absence of routine ovarian screening.

**Practice implications:**

Thought needs to be given to how best to create and disseminate credible ovarian cancer symptom information materials.

## Introduction

Women with a gene mutation or family history that predisposes them to developing ovarian cancer (OC) require effective risk management strategies. An individual’s lifetime risk of OC is influenced by many factors, including age of relative at diagnosis, relationship to diagnosed family member (roughly 4% risk of women with one first degree relative with OC) and number of diagnosed relatives (up to 14% risk of multiple relatives diagnosed) [1–3]. Mutations in the BRCA1 gene are associated with a 40–60% lifetime risk of OC and mutations in BRCA2 with a 10–30% lifetime risk [4–6]. These risk estimates are particularly high when compared to the general population lifetime risk of 2% [7].

The only effective OC risk management strategy is risk-reducing salpingo-oophorectomy (RRSO), which involves the pre-emptive removal of healthy ovaries and fallopian tubes [8]. It is recommended after the age of 35 to high-risk women, which is challenging and difficult due to the loss of fertility, premature menopause, and associated quality-of-life issue and the need to undergo surgery [4, 9]. The effectiveness of OC screening is unproven and this option is therefore not routinely available in many countries [10, 11]. Consequently, women at increased risk who decide to not have, or delay RRSO have to rely on early diagnosis through symptom awareness and presenting to primary care as soon as possible until effective screening strategies or chemoprevention becomes available.

The health beliefs of women at increased risk of OC are important to consider because in the absence of organised screening, the needs that were previously met by participating in screening trials must be met in other ways. Health beliefs are attitudes and beliefs or convictions that an individual has towards a health behaviour [12]. Participating in screening studies has been described by women as a proactive approach to OC detection [13]. Women report gaining considerable reassurance and wellbeing from being part of a screening study, which was perceived as a safety net, despite women being told repeatedly that its value was unproven [14]. Women at high-risk, especially those who previously participated in OC screening studies, may therefore need to seek out other sources of support in order to replace the proactive approach to cancer detection and reassurance they previously gained through participation in screening. Sources of support are also important for women who have not previously been involved in screening studies, as these women may still need support to manage their risk.

Cancer survival can be improved by encouraging individuals to present earlier for medical advice regarding potential symptoms [15, 16]. OC was once referred to as the silent killer, with this phrase frequently used to describe what was believed to be an asymptomatic disease [17–19]. However, this has been discounted over recent years, with numerous studies highlighting that OC does indeed have symptoms (including bloating, difficulty eating/change in appetite, and pelvic/abdominal pain), which are present in various stages of the disease, and are not just limited to advanced disease [18, 20–23]. This transition from a silent killer to a symptomatic disease has also been reflected in policy and medical guidelines [24, 25]. Symptom awareness could therefore be a route to earlier diagnosis [26]. However, in order for symptom awareness to be utilised to improve cancer detection, an understanding of symptom awareness and presentation behaviour is needed. At risk populations for specific cancers may possess more knowledge about the disease, may have different health beliefs, such as increased worry, and may endorse different barriers as a result of their risk status and perceived risk [27]. Tools to promote cancer symptom awareness and encourage earlier anticipated presentation include educational materials in the form of leaflets, diaries, factsheets, pamphlets, videos, and posters [28]. However, little is known about how women at increased risk access and perceive this information in the context of OC. There are concerns that encouraging regular self-monitoring through educational materials, such as diaries and checklists, may lead to anxiety and unnecessary investigations [27, 29, 30]. The potential influences that perceived risk and worry may have on decisions to seek medical help for possible OC symptoms in women at increased risk of OC is therefore of interest. High levels of worry could lead to hyper-vigilance, and therefore, information and guidance to manage women’s expectations could be needed if a symptom awareness strategy is utilised. This behavioural pattern could be problematic not only in terms of the individual’s psychological well-being, but also the potential impact on primary care services if women are frequently visiting their GP.

The aim of the present study was to gain insight into women’s experiences and perceptions of OC symptom management in the context of living with a family history of OC, and the proactive or preventative behaviours they may adopt in the absence of routine OC screening. Semi-structured qualitative interviews were carried out with women at increased risk who have previously taken part in screening to explore influences on OC awareness and anticipated symptomatic presentation, perceived benefits and barriers to presentation, and the acceptability of symptom awareness as a risk management strategy. Interpretative phenomenological analysis (IPA) [31] aimed to explore personal lived experiences and how people make sense of these experiences [31] and was used to gain a rich understanding of how women’s personal experiences of OC shape their perceptions and beliefs about the disease and its management.

## Method

### Participants

Participants were a subset of women who had taken part in an earlier quantitative study of the determinants of anticipated presentation with OC symptoms [32] and prior to that a psychological evaluation of familial OC screening (PsyFOCS) among women at increased risk of OC [14]. Women were considered at increased risk because of their family history or genetic test results. The subset interviewed in the present study was purposively sampled to reflect a variety of ratings of what women would do if they experienced symptoms of OC (anticipated presenting immediately, delaying up to one week, or delaying for over three weeks) and levels of OC worry (according to the Cancer Worry Scale, range of worry from 3–15) [33]. The sample that the subsets were selected from had an average worry score of 6.2 (standard deviation 1.9, range 3–12) [32]. Geographical proximity was also considered due to the face-to-face nature of the interviews, and therefore, women who lived within two hours travelling distance of the Cardiff research base were invited to participate. In total, 20 women agreed to be interviewed out of 32 invited (65%). Recruitment stopped when eight interviews had been completed (25%) as it was considered that there was coherence in responses and the accounts given by participants could be analysed in sufficient depth to answer the research question [34].

### Procedures

Cardiff University School of Medicine Research Ethics Committee provided approval for this study. Participants were sent study invitation materials and once consent had been obtained, the researcher telephoned to answer any questions about the research study and arranged an interview time. At the time of the interview, participants completed a further consent form and were given the opportunity to ask questions. Semi-structured interviews were conducted in the participants’ own homes, with the content of the interviews informed by the topic guide. The main topics were OC symptom perceptions, experience of symptoms, and anticipated presentation in the presence of OC symptoms. Other topics that arose during interviews were explored through the use of probes and prompts. The interviews were audiorecorded and transcribed verbatim. Average length of the interviews was 35 min (range 21–61 min).

### Data analysis

Transcripts were analysed using IPA [31] by SS. Each anonymised transcript was read and re-read and was analysed with themes noted for each transcript. This process was repeated until all themes within transcripts had been identified and noted. Once the themes that represented the different thoughts and feelings about a particular topic had been identified, the themes were clustered into superordinate themes. Abstraction was then used to identify links between emergent themes and a name was given to each superordinate theme that reflected the themes within the cluster. Another researcher (KB) independently coded three transcripts to check the applicability of the code framework, with discrepancies resolved through discussion. The data were then entered into NVivo 8 qualitative analysis software package. Results are organised around themes that emerged from the transcripts [35]. Quotes presented in Table 2 and discussed in the following section represent examples of the identified themes with narrative accounts provided to show the participants’ shared experience [36]. In the table, insertions to clarify topic content are denoted by square brackets. The removal of irrelevant information within the quotes is denoted by “….”. The characteristics of each participant are presented in parentheses after each quote in the following order: participant number (p1–p8), anticipated time to presentation (immediate, one week, over three weeks) and ovarian cancer worry score (w3–w8).

## Results

### Sample characteristics

Nine interviews took place across England and Wales (*n* = 2 in South West England and *n* = 6 in South Wales). Sample characteristics are provided in Table 1. Participants were aged 41–77 years, were mostly educated up to secondary level, and had been in OC screening between one and 10 years.

Key themes that were identified included OC screening, personal and familial experiences, symptom monitoring behaviour, OC symptom information sources, personal barriers and facilitators, and system barriers and facilitators.

### Ovarian cancer screening

Previous participation in OC screening studies was noted as a source of reassurance. Women felt that through participation and frequent interaction with healthcare professionals, any potential health problem would be identified. Some women went on to discuss how they would not necessarily look out for bodily changes between scans or tests, because they believed that screening would detect anything of concern. This could suggest possible over-reliance on screening.

In the absence of routine OC screening, some women had asked their GP to continue sending them for tests, while others were paying privately for screening. This suggests that women have sought to gain the reassurance they previously got from screening by either presenting to their GP or paying for tests to be done privately.

### Personal and familial experiences

Memories and personal experiences of OC being diagnosed in family members were discussed. These experiences had a strong influence on their own perceptions of OC, in terms of both recognising symptoms and disease progression once diagnosed. Experiences of OC within the family influenced perceptions of personal OC risk. A fear of diagnosis often stemmed from these experiences; however, some women described how the negative memories of OC in relatives were a driving force, due to wishing to avoid the same experience themselves.

Genetic factors were a source of worry and also led to concerns about what family members might experience if they were to be diagnosed with OC. Again, these concerns often stemmed from familial experience with the disease and not wanting to put their family member through these experiences.

### Symptom monitoring behaviour

Women described the different ways that they monitored themselves in order to monitor possible symptoms of OC. Keeping fit and being aware of their bodies was frequently endorsed, with women describing how they felt this would help them notice any changes more easily. There was also discussion of how family members had been unable to spot symptoms, with this experience being a driving force for their own body monitoring.

In terms of aide memoires, different preferences and techniques were expressed for monitoring symptoms. Some said that they would make notes to help them keep track of any symptoms they experienced, with others explaining how they would just rely on their recall of symptoms.

### Ovarian cancer symptom information sources

The Internet was quoted as the most common source for OC information: *‘I’d go and Google it’ (p2, immediate, w6), ‘I’d look on the net (p6, immediate, w4)’.* Information was sought for a variety of reasons, with information described as important for general OC knowledge and for preparing for the future and as a point of reference before going to seek medical help for a specific concern.

Alternatively, some women discussed how they would avoid information-seeking due to worry about what they might find and concerns about the credibility of web-based information. OC information was described as a source of worry with feelings of being overwhelmed by facts and figures for the disease expressed. Information was often considered daunting.

### Personal barriers and facilitators

Potential barriers to symptomatic presentation were suggested, including the vague nature of OC symptoms. Difficulty in recognising potential symptoms was frequently mentioned in relation to age. Many women felt that as they were getting older, they were finding it harder to distinguish between what might be a symptom of OC and what was part of the natural ageing process. Pre-existing medical conditions were also discussed, as women found it hard to differentiate the symptoms of OC from those associated with benign conditions.

Symptoms were also endorsed as facilitators. Women considered symptoms and bodily changes to be very important, with the perception that being aware of their own bodies would make it easier to spot any changes. Once the symptoms or bodily changes had been noticed, women felt they would act quickly and seek medical advice. Personal risk status was a facilitator to presentation in the presence of symptoms, with women reporting they would act quickly due to their concerns relating to their risk status.

Self-confidence was described as making it easier to voice concerns to the GP. Confidence was often embedded in risk status, with some women describing themselves as being confident and assertive due to their increased risk. Information was noted as a source of increasing confidence. Women felt that having knowledge and information was important in helping them to prepare for consultations with health professionals.

### System barriers and facilitators

Difficulty accessing primary care and difficulties communicating with GPs were described. Anticipated problems with getting GP appointments or long onward referrals were also expressed. In addition to these perceived barriers to getting an appointment, barriers were also perceived in the actual appointment. There were concerns over the lack of time given for consultations and the negative attitudes of the GP. These were often embedded in experiences of the GP not being aware of their personal risk status, which often made it harder to discuss their concerns. This lack of awareness was described as both a source of frustration and an additional emotional burden as a result of re-living the past.

Conversely, having a GP who was aware of their risk status helped some women feel more confident in presenting and expressing their concerns. In such instances, there was a sense of relief in not having to reiterate their family history. Continuity of care was considered important, with feelings that seeing the same GP helps develop a relationship, which in turn helped make them feel comfortable in expressing any concerns.

## Discussion

A qualitative study has been presented that explored experience and perceptions of OC symptom awareness and anticipated presentation in the context of increased risk of OC. Perceived risk and OC worry were found to influence and shape women’s perceptions about OC and were exacerbated by personal experiences with the disease and screening experiences they have had as a result of their risk status. OC experience as a result of familial diagnosis was integral to shaping participants’ beliefs about the disease and anticipated presentation with symptoms. These experiences were discussed in terms of both facilitators and barriers to OC knowledge, anticipated presentation, and information seeking. Previous engagement with OC screening also shaped beliefs about symptoms, and in some instances led to neglect of symptom awareness due to over-reliance on privately paid for tests.

Findings highlight the need for clear, credible, and trustworthy information on OC symptoms. Provision of evidence-based information from credible sources, or signposting to such resources, could help reassure women that they are accessing accurate and trustworthy information. This is particularly important in light of many people turning to the internet in search of health information [37]. Concerns were expressed in the current study about the vast quantity and varying quality of information that can be accessed via the internet, and even those who endorsed the Internet as an information resource questioned the credibility of some of the information. Information about OC was perceived both positively and negatively and could therefore be viewed as a double-edged sword, demonstrating the equivocal nature of information and how it is perceived differently by different individuals. Some women felt empowered by reading information and wanted to be up to date with all information about OC.

Others felt that in light of their risk status, information could be overwhelming and would lead to increased worry about disease susceptibility and outcomes. In particular, women felt that facts and statistics relating to OC was a source of concern when looking for information, with this discouraging them from searching for information. This could reflect helplessness, because if women read survival statistics and feel the outcomes are poor they may feel there is no need to be proactive. This poses questions about what type of information should be included in information resources for women at risk for OC. Findings suggest there is particular need for education on available risk management options as the reported utilisation of medical tests as a source of reassurance and hope is potentially worrying due to the uncertainty surrounding the clinical effectiveness of OC screening [10, 11, 38]. This trust in ovarian cancer screening was similarly reported by Lancastle and Brain [39], where women felt reassured and less distressed as a result of putting faith in the effectiveness of screening. The trust that women are placing on screening tests could suggest that women need to be educated about the current opportunities available to them. If women are unclear of the opportunities available to them they should be encouraged to go and visit their GP or genetic counsellor to discuss their personal situation and options.

An information resource, such as a symptom awareness tool that addresses the needs of women at high-risk could be a way to provide OC information and also maintain psychological well-being. Participants discussed the importance of presenting with symptoms regardless of whether it turns out to be ovarian cancer. Similar findings were reported by Low and Waller [40], where participants felt that even if symptoms were not likely to be cancer, it was considered beneficial to present as it could lead to the detection of other conditions. The anticipated willingness to present with OC symptom concerns discussed in the present interviews could be viewed positively since women are anticipating engagement with the healthcare system; however, this could also lead to over-presentation as a result of hyper-vigilance [27, 41–43]. Hyper-vigilance can be harmful to psychological well-being, with breast cancer literature reporting high frequency of thoughts about breast cancer, high perceived risk, and frequent breast self-examination in those at increased risk of breast cancer [29, 44]. Emphasis on new and unusual bodily sensations was often paired with keeping fit and slim in the current study, suggesting that women may be trying to undertake risk management strategies that are within their personal control. This behaviour could also be deemed as a compensatory mechanism to attempt to control worrying thoughts about OC risk [42, 45]. It may therefore prove useful to explain to women what the symptoms are and to explain that they may need to monitor their symptoms for a certain amount of time in order for GPs to consider a diagnosis of OC. Similar approaches have been used in informational leaflets for breast self-examinations to detect breast cancer [46]. Symptom education could be a way to help women manage their worry levels and reduce the likelihood of over presenting. However, in order for symptom information to be provided in materials, clinical information needs to be considered in order to determine what symptom time frames should be advocated to women. If women present with symptoms too early this could lead to GPs dismissing their concerns. Increasing knowledge about symptoms and their nature could mitigate against women inevitably associating any symptom experience with OC. Perceptions of anticipated presentation often stemmed from experiences where family members had delayed presentation, or had not monitored bodily changes. Women discussed how they would not act in the same way and would instead be proactive and decisive in the event of noticing changes they attributed to possible OC. Familial experience is therefore integrated into cognitions and emotions for women when considering what to do about symptoms.

Confidence was described as a driving force to presentation, as women felt they had to be confident in order to interact with health professionals [47]. Some women reported they would read up on OC information prior to going to see their GP, to ensure that they were fully prepared to voice concerns. Information on what to do before a GP visit may be well received by women at increased risk and could be incorporated into such a tool in order to psychologically prepare women for the consultation. A symptom awareness tool could therefore be useful as an act of preparation for GP consultations [48]. Women also discussed how they found it an emotional burden to reiterate their risk status, and thus, it may be useful to prime them for such an event so that they can be prepared to disclose this information. A paper-based tool may help women feel more confident in presenting as it will enable the tool to be taken along to aid discussion in the consultation [47, 48].

## Limitations

Study participants were selected based on anticipated presentation times, worry and geographical location. If a different sampling technique had been used, such as self-efficacy or age, a broader range of participants may have been included. However, it was felt that the sampling used was adequate and sufficient. The spread of anticipated presentation time was chosen to reflect the different presentation beliefs of the sample, with this reflecting the outcome of interest.

The potential lack of sample representativeness is also acknowledged, as the women were recruited from a pool of those who had participated in previous studies on OC symptom awareness and beliefs [14, 32]. These women had all also been part of ovarian cancer screening and therefore have different levels of ovarian cancer worry and symptom awareness than women who did not take part. Exploration of ovarian cancer worry and symptom awareness in women at increased risk of ovarian cancer but have not taken part in screening would be a useful next step for future research. Eight women participated, which could lead to questions concerning representativeness. However, it was felt that at this point in recruitment there was coherence in responses and that the accounts given by participants could be discussed in relation to the research question and could be extrapolated to relevant psychological theory [34]. It should also be acknowledged that while the participants were all at increased risk of OC, participants were likely to have a wide range of objective risk of OC, which could in turn influence perceptions about the disease. Therefore, future research should obtain information on individual risk status to enable understanding of OC perceptions in those at varying levels of increased risk.

## Practice implications

Education about symptomatic presentation and the current lack of effective screening for OC could be provided in a symptom awareness tool that has content specifically designed for women at increased risk. However, consensus regarding what symptom information should be given to women is crucial and could help manage expectations and maintain psychological well-being.

Thought needs to be given as to how OC information is disseminated. The Internet was frequently described as a controversial source of information due to issues with the credibility of information. While some women described how they would avoid information due to fears over content, it could be suggested that all women should be provided with access to credible, clear, and scientifically grounded information about OC. Support for this argument comes from the lack of understanding about the effectiveness of current screening options, confusion over OC symptoms, and when to act on them.

## Conclusions

The present study has explored how perceived risk and worry may influence decisions to seek medical help for possible OC symptoms in women at increased risk of OC. Women’s perceptions of OC and behavioural intentions relating to the disease have largely been shaped by their familial disease experiences and previous reliance on screening, and women may be cautious about searching for information independently. This stems from concerns over information quality and the content of some informational resources being overwhelming. There is therefore a need for reliable and clear information that does not lead to increased worry. Thought now needs to be given to how best to create and disseminate credible ovarian cancer symptom information materials.

## Conflicts of Interest

Authors Stephanie Smits, Jacky Boivin, Usha Menon, and Kate Brain declare that they have no conflict of interest.

## Figures and Tables

**Table 1.** Sample age, education, screening years, anticipated presentation, and ovarian cancer worry level.

Participant	Age	Education level	Years in ovarian screening	Anticipated presentation time	Ovarian cancer worry [33] (range 3–15)
1	70	Up to age 16	6	Immediately	3
2	77	Secondary	4	Immediately	6
3	56	Secondary	1	Up to 1 week	5
4	41	Degree and above	1	Up to 1 week	3
5	68	Secondary	10	Over 3 weeks	5
6	54	Degree and above	5	Immediately	4
7	50	Secondary	2	Immediately	7
8	44	Secondary	6	Over 3 weeks	8

**Table 2.** Illustrative quotes from interviews aligned to identified themes from interpretative phenomenological analysis^*^.

Theme	Description	Illustrative quote
Ovarian cancer screening	Comfort gained from screening	I think it’s just a comforting fact that you know that if something’s going to happen you know they’re going to do something about it and you can come from there thinking, well, everything’s fine. So it’s that feel good factor isn’t it. (p7, immediate, w7) I think, ‘Yeah, that’s me, I’m sorted, I’m okay,’ and then I think it’s time to have another one. And I do and I don’t think about it. (p8, over 3 weeks, w8)
	Continue to seek reassurance from screening	I have scans, ultrasound scans every year. I pay for them now because they used to be part of the study that I was taking part in and then that all stopped and the funding stopped and everything. So I thought I’d keep that up… so that’s my way of sort of keeping my eye on things. (p8, over 3 weeks, w8) I would rather be too cautious than dead, basically. I know that sounds really dramatic but I do keep on until, you know, I would rather keep on and keep on and keep on and say, look there’s nothing, rather than leave it lie and then say ‘Oh look, we’ve got a problem now’, which sometimes it’s too late isn’t it. (p7, immediate, w7)
Personal and familial experiences	Influence on perceptions	I suppose because of my sister having no symptoms… (p2, immediate, w6) … ‘cause I know when my cousin got it, she didn’t even realise, realise that she had it [ovarian cancer]. (p5, over 3 weeks, w5)
	Experience influencing anticipated presentation	… having gone through it all [with Mother and Grandmother who had ovarian cancer] I know roughly what to look for and if there’s anything amiss then to go and find out straightaway. (p1, immediate, w3) I mean if you’ve been down the road before, you know… you just wouldn’t waste time, would you, because time is precious. (p6, immediate, w4)
	Worry	I don’t want my children to go through that. Obviously they went through it with my mum, but I don’t want them to go through it with their mum. (p7, immediate, w7)
Symptom monitoring behaviour	Body monitoring	I know I don’t look fit, but I go to the gym three times a week, I’m careful of what I eat, you know… we know our bodies so well, we know what we are doing to them. (p6, immediate, w4) I think that’s what happened with my grandmother and her two sisters. They were all big ladies; they didn’t see what going on inside them and it was all kinds of things, and they died of it, you know. So I think it’s because they couldn’t really see the physical changes... I’m sort of fairly slim across my belly, so I think I’d notice bloating. I know one of the ones was bloating that doesn’t go down, is it? I think I’d probably notice that, but with a lot of women that may be bigger, they wouldn’t actually see that. (p8, over 3 weeks, w8)
	Monitoring techniques	I’d probably not make a diary, but make some notes of what my symptoms had been, where and when, yeah make a bit of a diary. (p4, 1 week, w3) I’d just sort of store it in my head probably, and then just go to the GP with general, you know general sort of symptoms and things rather than specific. (p8, over 3 weeks, w8)
Ovarian cancer symptom information sources	Information is useful	I think any information is a good thing isn’t it, prevention is better than cure…whatever information is quickly absorbed, because if it doesn’t apply to you, it could apply to another member of your family or a friend or something which could be useful. So I think any information is good whether it’s something you already knew or something new that you’ve learned. (p7, immediate, w7) If you want to check [the internet] that before you go to your GP, just ‘cause you think you’re worrying about nothing, you know, it’s private. (p8, over 3 weeks, w8) It could put some people off if they knew the statistics for success rates, survival rates. (p2,immediate, w6) …you can get rogue information and you can think, ‘Oh that’s alright then, they told me on the internet there’s no need to do so and so’, which if you had gone to the GP… he could have done something or helped something. (p7, immediate, w7)
	Information is a source of worry	…It can be overwhelming sometimes, you get too much information. (p3, 1 week, w5) ‘Cause too much information could really put that wheel in your head turning... because I just wouldn’t want to go down the road of just Googling the word and no, no, that would just put my head into override I expect. (p6, immediate, w4)
Personal barriers and facilitators	Symptom nature as a barrier	They call it the silent killer for one reason. All the symptoms are there but they don’t know about it. They think oh well, it could be a period, it could be this, it could be that, it could be what I’ve eaten. (p6, immediate, w4) I mean, any young person like you, if you started to get those then you would really start to worry, wouldn’t you? Someone my age, you say, well, okay I’ll get back ache and get wind a lot, you know, and I get changes in bowel movements so, you know they’re always changing. (p2, immediate, w6)
	Bodily changes as a facilitator	If I started to have any symptoms which I thought were unusual, I would immediately go to my doctor (p2, immediate, w6) If something was different from every day I would… I wouldn’t waste time. I’d go and get it checked straight away (p6, immediate, w4)
	Confidence as facilitator	I might be over, sort of worrying about it, and [thinking] if I haven’t actually got the symptoms, but because I know I’m at risk, I get symptoms, twinges, and I think ‘I wonder’ all the time. (p8, over 3 weeks, w8) It does make you a stronger person [experiencing family members with OC] because you get more determined I do believe, because you get more determined in your way. (p7, immediate, w7) If you’re armed with that information then the doctors can’t say ‘oh you’ll be all right, love, you know, it’s just a bit of ageing and diverticulitis or whatever’. If you actually know that information it’s easier to push. (p4, 1 week, w3)
System barriers and facilitators	GP appointment barriers	They haven’t got time to see anyone, have they? They don’t, they’re spending so much time on paperwork. (p5, over 3 weeks, w5)
	GP barriers	Some GPs don’t give a damn and others don’t know the information. (p4, 1 week, w3) They didn’t know anything about my family history at all. Okay, my file obviously would be that thick coming from when I was born, ‘cause they don’t have time to look at it do they? (p5, over 3 weeks, w5) You would have to go through it all [family history] and whatever… you’ve got to keep going through the same thing all the time. (p1, immediate, w3)
	GP facilitators	It’s better to see the same one as you don’t have to keep going through the same thing all the time. (p1, immediate, w3) It seems to give me peace of mind, because you don’t have to continually repeat all the time. (p7, immediate, w7)

* Insertions to clarify topic content are denoted by square brackets. The removal of irrelevant information within the quotes is denoted by “….”. The characteristics of each participant are presented in parentheses after each quote in the following order: participant number (p1–p8), anticipated time to presentation (immediate, one week, over three weeks) and ovarian cancer worry score (w3–w8).
